# Lightweight deep neural networks: Optimization of vehicle classification using ICBAM based on depthwise separable convolutions

**DOI:** 10.1371/journal.pone.0335967

**Published:** 2025-11-21

**Authors:** Qifeng Niu, Jinhui Han, Zhen Sui, Feng Xu

**Affiliations:** 1 School of Physics and Telecommunication Engineering, Zhoukou Normal University, Zhoukou, China; 2 College of Communication Engineering, Jilin University, Changchun, Jilin, China; Central South University, CHINA

## Abstract

Vehicle classification is a core task in intelligent transportation systems, where high demands are placed on both computational efficiency and generalization ability in practical applications. Existing deep learning models often struggle to meet these requirements due to their high computational complexity and limited generalization. To address this challenge, this study proposes a lightweight and efficient deep neural network called DSICBAMNet, which achieves high classification accuracy while significantly improving computational efficiency. The design of DSICBAMNet is centered on two key components: Depthwise Separable Convolutions (DSC) and an Improved Convolutional Block Attention Module (ICBAM). The DSC module reduces the number of parameters and computational complexity by decomposing convolution operations, making it well-suited for resource-constrained deployment scenarios. Meanwhile, ICBAM addresses the shortcomings of traditional CBAM in terms of overfitting resistance and feature weighting strategies. By introducing Dropout regularization into the channel attention module, ICBAM enhances the model’s resistance to overfitting. Additionally, it optimizes the interaction mechanisms and weight distribution between the channel and spatial attention modules, enabling more accurate multi-class feature representation. The network achieves efficient multi-scale feature extraction by stacking multiple improved DSICBAM blocks while maintaining an overall lightweight structure. In experimental evaluations, DSICBAMNet was compared with five classic models, including AlexNet and MobileNetV2. Experimental results demonstrate that DSICBAMNet achieves outstanding performance on both the MIO-TCD dataset, with 286 test samples and an average classification accuracy of 97.36%, and the Stanford Cars dataset, with 1,060 test samples and an accuracy of 96.51%. Moreover, the combination of Grad-CAM visualizations and confusion matrix analysis validates the model’s ability to focus on key regions and maintain consistency in classification outcomes. These results underscore the model’s potential applicability and practical value in intelligent transportation scenarios.

## Introduction

Vehicle classification is pivotal in intelligent transportation systems, driving advancements in traffic management, autonomous driving, and public safety monitoring [1–3]. Accurate vehicle identification enables efficient traffic flow analysis, road planning, violation detection, and accident prevention [4,5]. However, diverse vehicle types, visually similar appearances, and complex environments—such as low-light conditions and dynamic backgrounds—pose significant challenges [6–8]. Traditional methods often underperform in such scenarios. While deep learning has achieved notable success in classification tasks, its high computational demands, resource requirements, and limited generalization hinder deployment in resource-constrained environments like edge devices and traffic monitors [9,10]. These challenges can compromise traffic efficiency, elevate accident risks, and lead to economic and societal repercussions [11,12]. Thus, developing a lightweight, efficient, and accurate vehicle classification model is crucial for modern intelligent transportation systems. Such a model would enhance adaptability, reduce costs, and support the evolution of smarter and more sustainable traffic solutions.

Traditional vehicle classification methods often rely on manually designed features and rules combined with classifiers [13,14]. While interpretable in specific scenarios, these methods lack flexibility and adaptability, limiting their effectiveness in complex environments. They are also less robust to variations in lighting, angles, and backgrounds, making them unsuitable for practical applications. Deep learning has emerged as a superior alternative, offering end-to-end feature extraction and rich representational capabilities. Unlike traditional methods, deep learning models can automatically learn features from data, significantly improving classification accuracy and robustness. Standard Convolutional Neural Networks (CNN) achieve better feature representation by deepening the network through additional convolutional and fully connected layers. However, increasing the number of filters in convolutional layers can improve channel learning but also escalates computational costs. To address this, researchers have explored reducing filter sizes or employing decomposition techniques, which split a single convolution operation into two sequential convolutions, effectively reducing computational demands without compromising performance. Shvai et al. [15] combined simple CNN with manually extracted labels for vehicle identification, enhancing practicality and accuracy in real-world applications. Balasubramaniam et al. [16] applied LeNet with corrected Rectified Linear Unit (ReLU) for breast image detection, improving representation of complex medical images. Ismail et al. [17] enhanced AlexNet by increasing layers and introducing ReLU, achieving exceptional performance in time-series classification on large datasets. Sengupta et al. [18] developed a hybrid model combining VGG and residual networks (ResNet), balancing accuracy and convergence speed for complex image classification tasks. Wen et al. [19] transformed time-domain signals into image data and optimized ResNet50 using a transfer learning framework, demonstrating excellent cross-dataset learning capabilities. Gulzar et al. [20] customized MobileNetV2 with five additional layers, achieving lightweight and effective fruit classification. Donuk et al. [21] utilized VGG-11 for facial expression recognition, pioneering the direct inference of human emotions from facial features. Kaur et al. [22] proposed the Deep Skip Connection Network or leukemia diagnosis, integrating skip connections and regularization techniques to achieve high diagnostic accuracy. Cao et al. [23] combined the Convolutional Block Attention Module (CBAM) with VGG for emotion recognition, effectively capturing key facial features and generalizing well across datasets. Shakibhamedan et al. [24] introduced an energy-efficient ACE-CNN classification algorithm, leveraging approximate multipliers to improve computational efficiency and accuracy. Tatsunami et al. [25] enhanced Long Short-Term Memory (LSTM) models with Sequencer modules, excelling in feature extraction and adaptability for complex tasks. Goerttler et al. [26] proposed a lightweight CNN architecture integrating multi-scale processing and attention mechanisms, using complementary pooling to optimize computational efficiency. Bhardwaj et al. [27] combined ResNet and U-Net to leverage ResNet’s deep feature extraction and U-Net’s precise localization capabilities, with augmented data preprocessing enhancing robustness. Gawali et al. [28] integrated CNN and recurrent neural networks for image sequence analysis, effectively capturing spatial-temporal dependencies and improving computational efficiency with a novel feature reduction strategy.

In recent years, deep learning has advanced vehicle classification, but existing methods face key limitations. Classical models like ResNet offer high accuracy but demand heavy computation and memory, making them impractical for resource-limited deployments. MobileNet [29], while lightweight with depthwise separable convolutions, struggles in complex backgrounds, varying lighting, and multi-scale feature extraction. Traditional attention mechanisms [30–32], such as CBAM, improve feature focus but often fail to optimize weight distribution, reduce redundancy, and prevent overfitting, limiting effectiveness in multi-class tasks and diverse datasets.

Moreover, many models rely heavily on large labeled datasets, posing challenges when annotated data is scarce or when encountering unseen vehicle variations, angles, and environments. Their limited generalization to real-world scenarios with occlusions, dynamic backgrounds, and low light remains a major drawback. Additionally, most lack the efficiency and low latency needed for real-time applications on edge devices with constrained resources. Poor interpretability further hinders deployment in safety-critical areas like autonomous driving and traffic monitoring.

To address these challenges, this study introduces DSICBAMNet, a lightweight and efficient deep learning model specifically designed to improve the practicality and generalization of vehicle classification tasks. DSICBAMNet adopts depthwise separable convolutions to significantly reduce parameter counts and computational complexity, ensuring compatibility with resource-constrained deployment scenarios such as edge computing and mobile devices. The model also features an enhanced attention mechanism that improves the interaction between channel and spatial features while optimizing weight distribution. This design not only strengthens the model’s robustness against interference but also boosts its capacity for multi-class feature learning. Additionally, DSICBAMNet integrates a multi-scale feature extraction module to adapt effectively to complex backgrounds and dynamic scenes. These improvements collectively ensure superior real-time performance and robustness in practical vehicle classification tasks, offering advancements in computational efficiency and classification accuracy.

The main contributions of this paper are as follows:

(1) A lightweight deep learning model, DSICBAMNet, is proposed based on depthwise separable convolutions and an improved attention mechanism. This model significantly reduces computational complexity and is well-suited for resource-constrained application scenarios.

(2) Building upon the traditional CBAM, Dropout regularization is introduced to enhance overfitting resistance. Additionally, the interaction mechanisms and weight distribution strategies of the channel and spatial attention modules are optimized, strengthening the model’s ability to capture multi-class features.

(3) By stacking multiple improved DSCBAM blocks, the model’s capability to extract features from complex scenes and multi-scale features is enhanced, while maintaining a lightweight design.

(4) Through various experiments, DSICBAMNet outperforms comparative models in both classification accuracy and computational efficiency, thoroughly validating its practicality and robustness for real-world vehicle classification tasks.

## Methodology

### Depthwise separable convolution

Traditional convolution extracts features by simultaneously processing spatial and channel dimensions to build complex representations. However, its computational complexity increases rapidly with the growth of input/output channel numbers and kernel sizes. While this method can capture rich feature information, its high computational cost makes it unsuitable for practical deployment in resource-constrained environments such as mobile devices and embedded systems. Additionally, traditional convolution, while performing both spatial feature extraction and channel fusion, tends to introduce computational redundancy, especially when handling high-resolution images.

To address these issues, depthwise separable convolution was introduced. It decomposes the traditional convolution into two independent steps: spatial filtering followed by channel fusion. This approach significantly reduces the number of parameters and computational load, while still maintaining excellent feature extraction capabilities. By separating the tasks of spatial feature extraction and channel fusion, depthwise separable convolution optimizes computational efficiency, making it ideal for environments where computational resources are limited, such as mobile and embedded systems.

In depthwise convolution, the convolution operation is applied independently to each of the three channels of the vehicle image. This means that each channel is filtered using a convolutional kernel of size M×M, and the information between the channels is not mixed. Let’s assume the feature map of the input data is denoted as *K*, with a size of P×L, the number of input channels is *Q*_*in*_, and the number of output channels is *Q*_*out*_. Each channel applies its own convolution kernel to generate the corresponding output $\psi_{d}$. The depthwise convolution parameter count $\text{Parameter}{\text{s}}_{D}$ can be expressed using the following formula:

$$\psi_{d}=K*W_{d}\text{, }W_{d}\in {\text{R}}^{M\times M\times Q_{in}}$$ (1)

$$\text{Parameter}{\text{s}}_{D}=M\times M\times Q_{in}$$ (2)

The total computational cost $\text{Computatio}{\text{n}}_{F}$ for the depthwise convolution operation can be expressed as the following:

$$\text{Computatio}{\text{n}}_{F}=M\times M\times Q_{in}\times P\times L$$ (3)

In pointwise convolution, a convolution kernel of size 1×1 is applied to each position in the input feature map. The kernel operates across all input channels and generates the output values for the output channels. Each 1×1 -sized convolution kernel performs weighted computations only along the channel dimension, without changing the spatial locations. Pointwise convolution performs feature fusion $\psi_{p}$ along the channel dimension with a kernel size of 1×1, and the total number of parameters $\text{Parameter}{\text{s}}_{G}$ in this process can be expressed by the following formula:

$$\psi_{p}=\psi_{d}*W_{p}\text{, }W_{p}\in {\text{R}}^{1\times 1\times Q_{in}\times Q_{out}}$$ (4)

$$\text{Parameter}{\text{s}}_{G}=Q_{in}\times Q_{out}$$ (5)

The total computational cost $\text{Computatio}{\text{n}}_{H}$ for the pointwise convolution process can be expressed as:

$$\text{Computatio}{\text{n}}_{H}=Q_{in}\times Q_{out}\times P\times L$$ (6)

Based on the above, we can derive the total parameter count $\text{Parameter}{\text{s}}_{total}$ and computational cost $\text{Computatio}{\text{n}}_{total}$ for depthwise separable convolution, which can be expressed as follows:

$$\text{Parameter}{\text{s}}_{total}=M\times M\times Q_{in}+Q_{in}\times Q_{out}$$ (7)

$$\text{Computatio}{\text{n}}_{total}=M\times M\times Q_{in}\times P\times L+Q_{in}\times Q_{out}\times P\times L$$ (8)

The parameter count for traditional convolution is $M\;\times \;M\;\times \;Q_{in}\;\times \;Q_{out}$, and the computational cost is $M\;\times \;M\;\times \;Q_{in}$ $\times \;Q_{out}\;\times \;P\;\times \;L$. In contrast, depthwise separable convolution effectively reduces both the parameter count and computational complexity.

### Improved attention mechanism

The traditional CBAM module typically relies on fully connected layers to directly learn global features when generating channel attention weights. While this method captures overall information, it is prone to overfitting in small sample sizes or complex scenarios, limiting the model’s generalization ability. Additionally, the sequential operation of calculating “channel attention” and “spatial attention” may result in the loss of key information during the information transfer process, preventing the spatial attention module from fully utilizing effective features across channels.

To address these issues, we introduce Dropout regularization in the channel attention module. By randomly dropping some neurons, the model’s resistance to overfitting is enhanced. Moreover, we propose a parallel fusion-based interaction mechanism, where both channel and spatial attention are calculated simultaneously, while learnable parameters dynamically adjust their fusion ratio. This optimizes the information exchange and weight distribution between the attention modules, allowing the model to capture multi-category features more flexibly. As a result, the overall accuracy and robustness of feature extraction are significantly improved.

In this process, we set the input data features as *E*, which is in a three-dimensional data format Q×W×A. In the earlier stage of this module, the data will pass through a global average pooling layer and a global max pooling layer. The outputs of both layers are then combined as two sets of feature dimensions for evaluation. This can be expressed as:

$$E_{\text{avg}}=\frac{1}{W\times A}\sum_{i=1}^{W}\sum_{j=1}^{A}E(i,j)$$ (9)

$$E_{\max}=\underset{i,j}{\max}E(i,j)$$ (10)

Where $E_{\text{avg}}$ and $E_{\max}$ correspond to the output results of global average pooling and global max pooling, respectively.

In the next process, the results from Eqs (7) and (8) will be integrated to achieve a concatenated result. To address the issue of information loss during the concatenation process, a Dropout regularization mechanism is added to the fusion module in this paper. This can be expressed as:

$$G=\delta (\text{Dropout(}W_{1}\cdot F_{avg}\text{ + }b_{1}\text{)})$$ (11)

where *W*_1_ is the weight vector of the first fully connected layer, *b*_1_ is the corresponding bias vector, and *δ* is the corresponding ReLU activation function.

Then, the data passes through the second fully connected layer, which can be expressed as:

$$M_{c}=\sigma (W_{2}\cdot G+b_{2})$$ (12)

where *W*_2_ is the weight vector of the second fully connected layer, *b*_2_ is the corresponding bias vector, *σ* is the corresponding Sigmoid activation function, and *M*_*c*_ is the generated channel weight output.

To overcome the insufficient information utilization between the interaction modules in the sequential operation mode, we propose a parallel fusion-based interaction mechanism. This simultaneously optimizes the weight distribution strategy of the channel and spatial attention modules, allowing the model to more flexibly capture multi-category features. The specific computation can be expressed as:

$$M=\alpha \cdot M_{c}+\beta \cdot M_{s}$$ (13)

where *M*_*c*_ is the channel attention weight, *M*_*s*_ is the spatial attention mechanism, *α* and *β* are the learnable parameters for dynamic adjustment, satisfying α+β=1, and *M* is the final attention weight generated from the fusion.

## ICBAM vehicle classification model based on depthwise separable convolution

**Fig 1:**
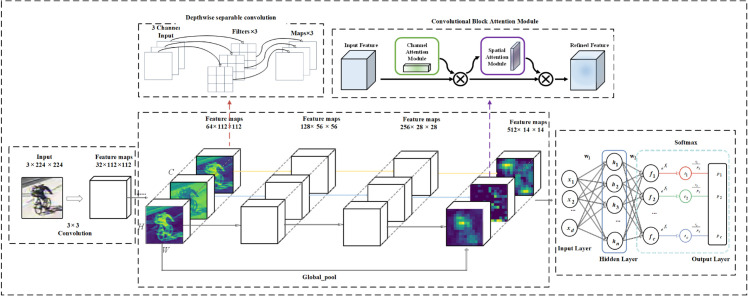
Vehicle classification model based on depthwise separable convolution and ICBAM.

As shown in Fig 1, we propose a DSICBAM-based model designed specifically for vehicle classification tasks, with a focus on efficiency, lightweight architecture, and generalization ability. The model primarily consists of a feature extraction module, an improved attention mechanism module (ICBAM), and a classification module. The feature extraction module employs depthwise separable convolution, which decomposes traditional convolution into depthwise convolution for spatial dimensions and pointwise convolution for channel dimensions. This approach significantly reduces computational complexity and the number of parameters, making it suitable for resource-constrained scenarios while enhancing feature extraction accuracy. The choice of depthwise separable convolution is based on its excellent performance in various lightweight networks and its ability to capture multi-scale features of vehicles effectively.

The improved CBAM module (ICBAM) integrates Dropout regularization into the traditional CBAM framework to alleviate overfitting in complex environments and enhance the model’s generalization ability. Additionally, it employs a parallel fusion structure of channel attention and spatial attention, combined with a dynamic weight allocation strategy. This enhances the expression of multi-class vehicle features and ensures sufficient feature interaction, improving the model’s adaptability to complex backgrounds and interferences. The classification module maps the extracted significant features into vehicle categories through fully connected layers and the Softmax activation function, achieving highly accurate classification decisions. By collaboratively optimizing each module, the overall model achieves efficient feature extraction, refined attention mechanism optimization, and enhanced classification performance while maintaining low computational complexity and robust generalization. This makes it suitable for real-time applications in practical vehicle classification tasks.

To further clarify the model structure, Table 1 provides a detailed breakdown of the parameters for each layer. The initial convolution layer (Conv1) uses a 3×3 kernel with a stride of 2 and padding of 1, mapping the input to dimensions (32,112,112). The purpose of the initial convolution layer is to quickly extract low-level features from the input image and perform preliminary downsampling to reduce computational load. The 3×3 kernel balances the receptive field and efficiency, while padding of 1 maintains the feature map dimensions. Setting the output channels to 32 ensures basic feature representation capacity while controlling model complexity, laying the groundwork for subsequent layers to increase channels (64, 128, 256, 512), thereby promoting multi-level feature extraction and improved classification performance.

**Table 1:** Model parameters in this article.

Network layer	Convolution kernel size	Stride	Filling number	Output
Conv1	(3, 3)	(2, 2)	(1, 1)	(32, 112, 112)
DSICBAMBlock 1	(3, 3)	(1, 1)	(1, 1)	(64, 112, 112)
DSICBAMBlock 2	(3, 3)	(1, 1)	(1, 1)	(128, 56, 56)
DSICBAMBlock 3	(3, 3)	(2, 2)	(1, 1)	(256, 28, 28)
DSICBAMBlock 4	(3, 3)	(2, 2)	(1, 1)	(512, 14, 14)
Global_pool	-	-	-	(512, 1, 1)
FC	-	-	-	(11)

*Table notes:* This table describes the parameters used in the model, including convolution kernel size, stride, and output dimensions.

The model subsequently incorporates four DSICBAM modules with output channels of 64, 128, 256, and 512, respectively. This design is inspired by the hierarchical structures of lightweight networks such as ResNet and MobileNet. Combined with experimental tuning, the four-layer structure effectively balances model depth, computational load, and overfitting risks. Notably, in the third and fourth DSICBAM modules, downsampling is achieved through convolution with a stride of 2, further extracting deep semantic information, compressing feature map dimensions, and enhancing the model’s representational and computational efficiency. Finally, the model uses global pooling (Global_pool) to compress features into dimensions (512,1,1), followed by a fully connected layer (Fc) to output 11 vehicle categories. This hierarchical design balances multi-scale feature representation with model lightweightness, achieving efficient and accurate vehicle classification.

### Introduction to vehicle dataset

**Fig 2:**
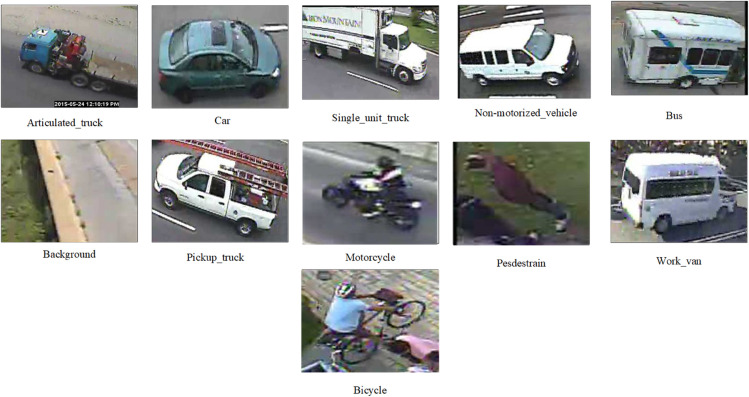
Partial vehicle dataset images.

In this study, the MIO-TCD Classification Dataset was selected as the experimental foundation. Specifically designed for vehicle classification tasks in traffic scenarios, this dataset comprises diverse vehicle types captured in real-world traffic environments, including cars, trucks, buses, motorcycles, and more, encompassing a total of 11 categories, as illustrated in Fig 2. To ensure diversity and representativeness, we randomly sampled 1,430 images from the dataset, striving for a balanced distribution across categories, with an average of 130 images per category. This sampling strategy allows the model to effectively learn distinguishing features across different vehicle types, thereby enhancing classification performance and generalization capability. For dataset partitioning, an 8-to-2 ratio was employed: 80 percent of the images, totaling 1,144, were used for training the model, while the remaining 20 percent, or 286 images, constituted the test set to evaluate model performance. The sampling and partitioning processes adhered to random principles while maintaining consistent category distributions, ensuring high-quality, balanced samples for both training and testing purposes. This division ensures that the model is trained with sufficient data while also allowing for an objective evaluation of the model’s generalization ability on unseen data. It is worth mentioning that the MIO-TCD dataset not only features high levels of image quality and annotation accuracy, but also presents challenges such as lighting, occlusion, angle, and complex backgrounds, since the images are captured from real traffic scenes. This provides a rigorous and practically significant testing platform for the model. With this strict data selection and partition strategy, we are able to comprehensively and accurately evaluate the performance and robustness of the proposed method in vehicle classification tasks, providing a solid data foundation for the deployment of real-world intelligent transportation systems. The specific dataset is shown in Table 2:

**Table 2:** Vehicle data and recognition labels.

Vehicle Type	Data Format	Training Set Samples/Individual	Test Set Samples/Individual	Recognition Label
Articulated_truck	(3, 224, 224)	104	26	0
Bicycle	(3, 224, 224)	104	26	1
Bus	(3, 224, 224)	104	26	2
Car	(3, 224, 224)	104	26	3
Motorcycle	(3, 224, 224)	104	26	4
Non-motorized_vehicle	(3, 224, 224)	104	26	5
Pedestrian	(3, 224, 224)	104	26	6
Pickup_truck	(3, 224, 224)	104	26	7
Single_unit_truck	(3, 224, 224)	104	26	8
Work_van	(3, 224, 224)	104	26	9
Background	(3, 224, 224)	104	26	10

*Table notes:* This table lists the details of vehicle types, their data formats, and recognition labels.

### Data preprocessing

In this study, to enhance the model’s performance in vehicle classification tasks, we implemented a series of meticulous data preprocessing operations on the raw image data. First, the original PIL image was converted into a NumPy array, and the image format was changed from RGB to BGR using OpenCV. This was done because many image processing functions in OpenCV perform more efficiently in the BGR format. Next, we applied the non-local means denoising algorithm by calling OpenCV’s fastNlMeansDenoisingColored function with parameters h = 10, hColor = 10, templateWindowSize = 7, and searchWindowSize = 21 to denoise the image.

Non-local means filtering effectively removes random noise while preserving edges and fine details, avoiding the blurring issues commonly associated with traditional mean filtering. Compared to other denoising methods, it adaptively weights pixel information based on similarities within image regions, achieving an optimal balance between noise suppression and detail retention. Furthermore, this algorithm strikes a favorable compromise between effectiveness and computational efficiency, making it suitable for rapid processing of large-scale image datasets. After denoising, the images are converted back to RGB format to meet the input requirements of the model. This preprocessing workflow significantly enhances image quality and feature representation, providing a robust data foundation for accurate classification in subsequent model stages.

## Experimental results and analysis

In this experiment, we built a deep learning experimental environment based on Python and PyTorch, providing a solid technical foundation and excellent reproducibility for the entire experimental process. Our system uses an Intel i5-13600KF processor and an NVIDIA GeForce RTX 4050 graphics card, equipped with 2560 CUDA cores and 6GB of VRAM, utilizing CUDA acceleration for large-scale data processing and deep network training. The parallel computing capability of the GPU significantly reduced model training and inference time, enabling efficient computation even when handling complex model architectures (such as the model combining depthwise separable convolutions and the improved CBAM module). With the powerful distributed computing support and automatic differentiation features of PyTorch 2.0+, we were able to better fine-tune the model’s hyperparameters and achieve fast convergence, ensuring excellent performance in the vehicle classification task. The collaboration between hardware configuration and software environment provided strong support for the model’s efficiency, accuracy, and robustness in this study.

### Comparative experiment

In this study, to validate the superiority of the proposed DSICBAM model in vehicle classification tasks, we selected five models for comparison: CNN [33], AlexNet [34], VGG-11 [35], MobileNetV2 [36], and DSCNet [37]. The traditional CNN model has a simple structure and low computational complexity, but its feature extraction ability is limited, resulting in average classification accuracy. AlexNet improves generalization ability by introducing ReLU activation and Dropout regularization, but it has a large number of parameters and an outdated structure. VGG-11 enhances feature extraction with a unified 3×3 convolution kernel design, but its high computational complexity results in lower efficiency. MobileNetV2 uses depthwise separable convolutions and a linear bottleneck structure, offering higher computational efficiency, but its ability to capture complex multi-class features is limited. DSCNet combines depthwise separable convolutions with a multi-scale feature extraction module, achieving a good balance between efficiency and accuracy, but its ability to filter feature information still has room for improvement. In contrast, the DSICBAM model comprehensively optimizes the traditional CBAM attention mechanism by introducing Dropout regularization to mitigate overfitting, and optimizing the interaction mechanism and weight distribution strategy of the channel and spatial attention modules. This enhances the model’s ability to capture multi-class complex features and its robustness against interference.

Table 3 presents the performance comparison of various models on the test set. The results show that our proposed DSICBAM model significantly outperforms other comparison models in average accuracy (97.36%), Top-1 accuracy (97.23%), and Top-5 accuracy (98.20%). In contrast, traditional CNN and AlexNet models perform relatively weakly due to limited feature extraction capabilities, with average accuracies of 82.12% and 89.73%, respectively. While VGG-11 improves classification performance with a deeper network structure, its fixed-depth design leads to greater computational overhead. MobileNetV2 and DSCNet, as lightweight models, perform well with the support of depthwise separable convolutions, achieving average accuracies of 96.85% and 95.63%, respectively, but their feature extraction capabilities are somewhat limited in complex scenarios. In contrast, DSICBAM combines depthwise separable convolutions with an improved attention mechanism, significantly enhancing feature interaction and multi-class feature capture capabilities. Moreover, through regularization strategies, it effectively alleviates overfitting issues, demonstrating exceptional classification performance while maintaining low computational complexity and strong generalization ability, making it the best-performing solution among all comparison models.

**Table 3:** Testing results of various models.

Comparison Model	Average Accuracy (%)	Precision (%)	Recall (%)	F1-score (%)	Top1-acc (%)	Top5-acc (%)
CNN	82.12	80	84	82	80.45	87.90
AlexNet	89.73	88	91.5	89.75	88.32	92.85
VGG-11	91.23	90	92.5	91.25	90.56	94.21
MobileNetV2	96.85	95.5	98	96.75	96.21	97.83
DSCNet	95.63	94.5	96.75	95.62	95.07	96.52
DSICBAM (Ours)	97.36	96.5	98	97.25	97.23	98.20

*Table notes:* This table presents the testing results of various models, comparing average accuracy, precision, recall, F1-score, Top-1 accuracy, and Top-5 accuracy. The proposed DSICBAM model achieves the highest performance across all metrics.

In the evaluation of Precision, Recall, and F1-score, DSICBAM still performs excellently, exhibiting high Precision and Recall values, reflecting its ability to accurately capture class features in classification tasks. Especially in complex tasks and imbalanced data environments, DSICBAM’s F1-score far exceeds that of other comparison models, proving its high stability and effective support for multi-class classification.

**Fig 3:**
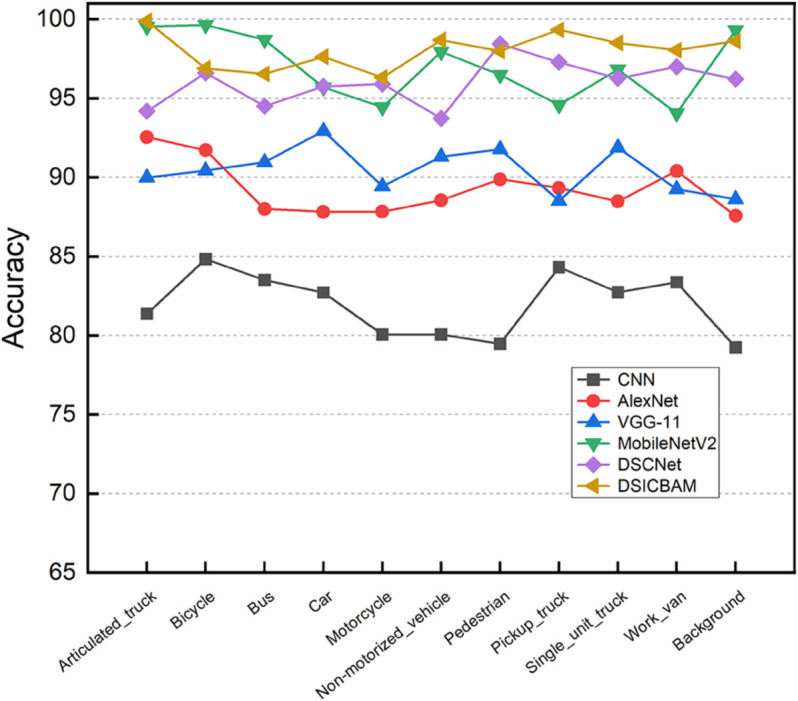
Comparison of accuracy across different categories for various comparison methods.

**Fig 4:**
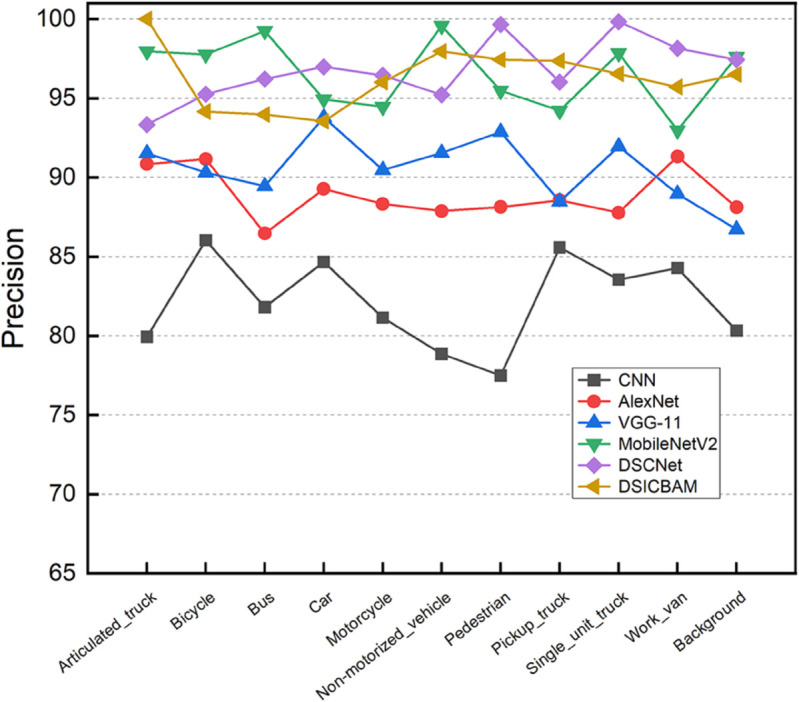
Comparison of precision across different categories for various comparison methods.

**Fig 5:**
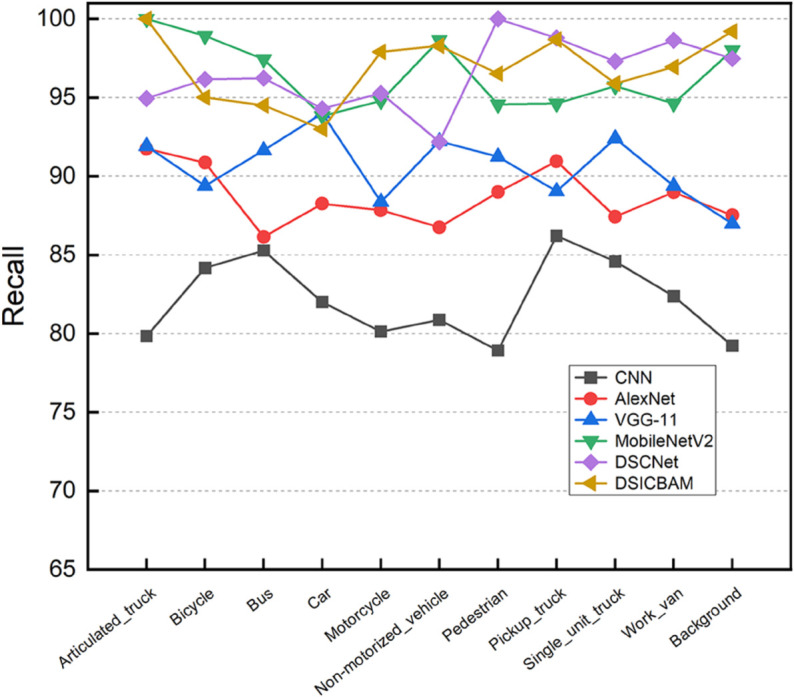
Comparison of recall across different categories for various comparison methods.

**Fig 6:**
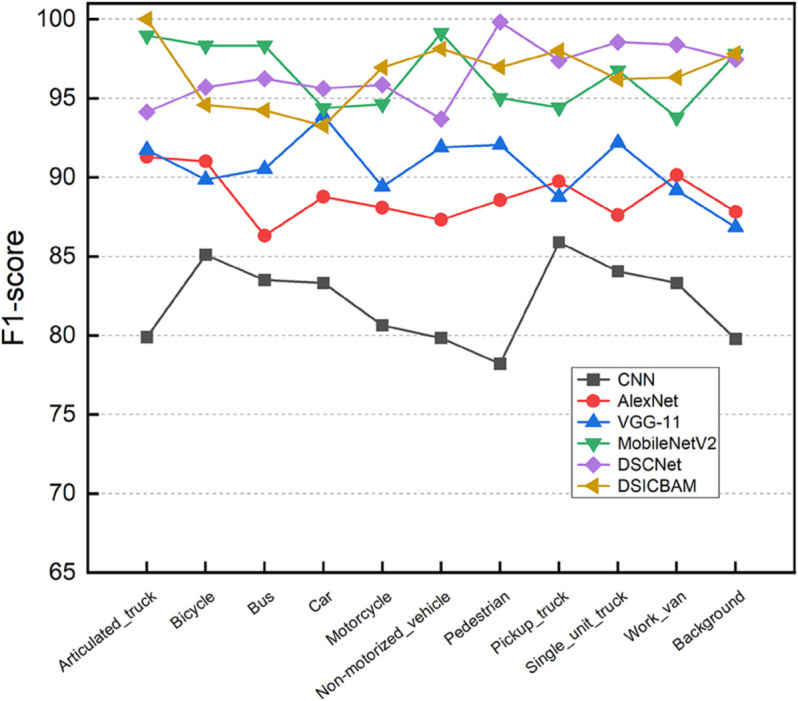
Comparison of F1-score across different categories for various comparison methods.

Fig 3, Fig 4, Fig 5, and Fig 6 show the comparison of accuracy, precision, recall, and F1-score across different categories for various models. It is clear from the figure that DSICBAM significantly outperforms other models in all metrics, especially in some complex categories, which further demonstrates its powerful feature extraction and classification capabilities.

**Fig 7:**
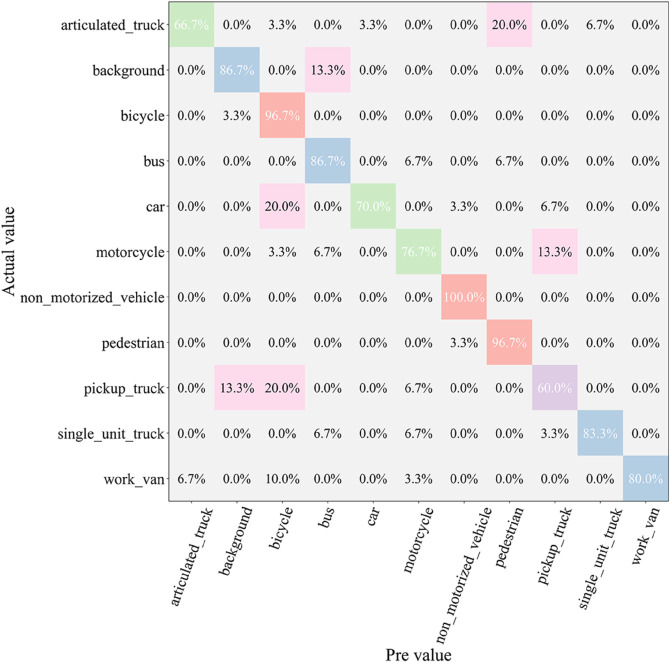
Confusion matrix results of different models-CNN.

**Fig 8:**
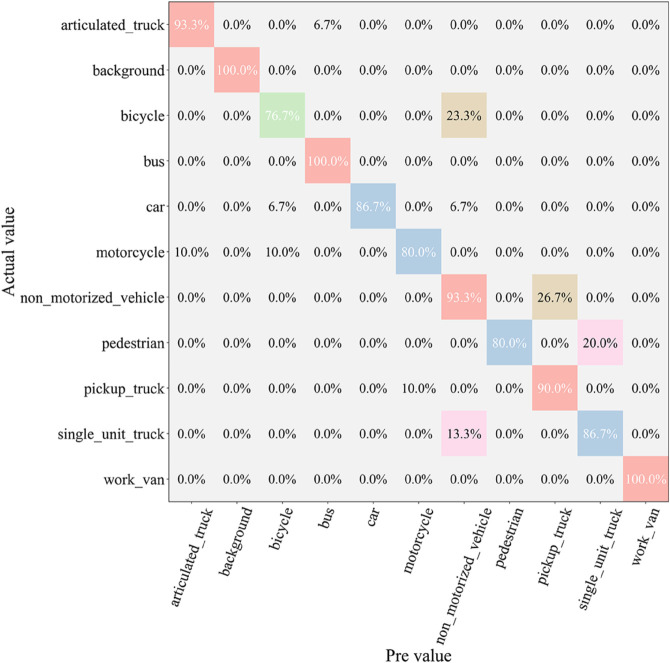
Confusion matrix results of different models-AlexNet.

**Fig 9:**
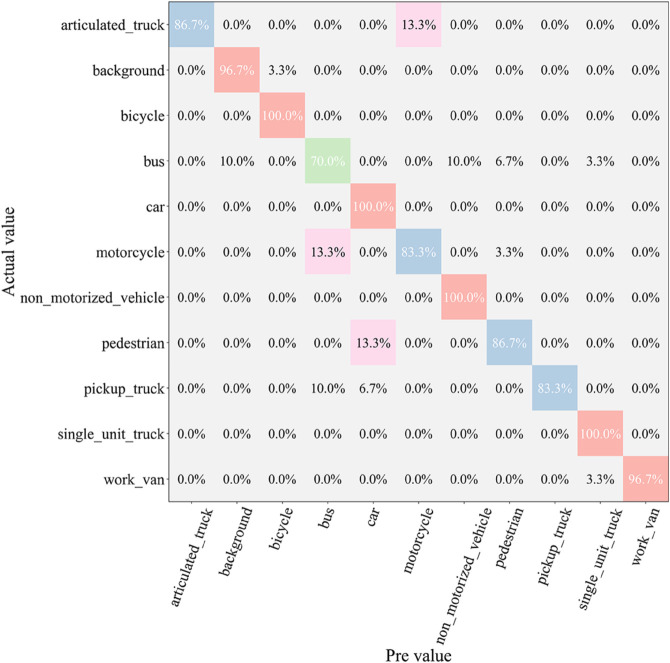
Confusion matrix results of different models-VGG-11.

**Fig 10:**
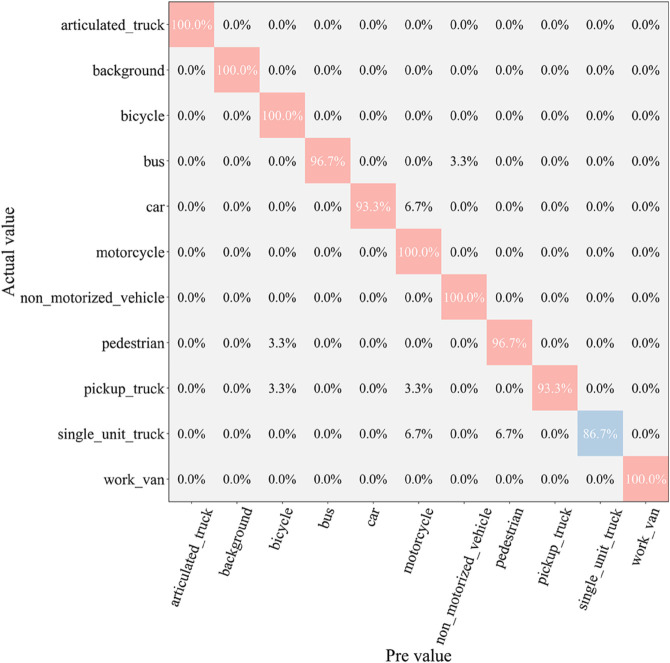
Confusion matrix results of different models-MobileNetV2.

**Fig 11:**
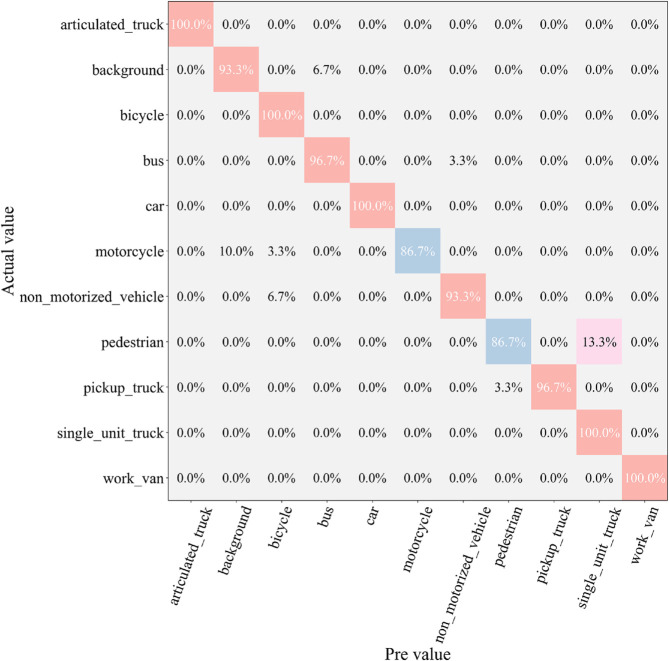
Confusion matrix results of different models-DSCNet.

**Fig 12:**
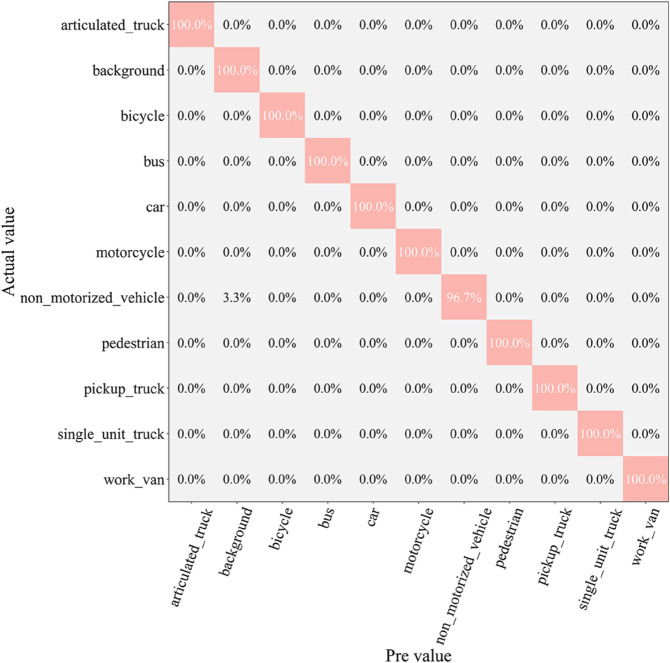
Confusion matrix results of different models-DSICBAM.

After the testing phase, to comprehensively assess the classification ability of each model across different vehicle categories, we generated a confusion matrix, as shown in Fig 7, Fig 8, Fig 9, Fig 10, Fig 11, and Fig 12. Combined with the data in Table 3, the analysis reveals significant differences in classification performance among the models.

First, the confusion matrices for the traditional CNN and AlexNet models show noticeable inter-class confusion. These models exhibit a high misclassification rate for vehicle categories with similar features. This is mainly due to their limited feature extraction capabilities, which make it difficult to capture subtle differences between categories. This limitation directly leads to lower average accuracy (82.12% and 89.73%) and Top-1 accuracy (80.45% and 88.32%). VGG-11 shows a significant improvement in classification performance compared to the traditional models. Its deeper network structure enhances feature extraction capabilities, resulting in a more balanced performance in the confusion matrix. However, due to the computational overhead and model depth, there are still some misclassification issues for categories with complex or highly similar features. The Top-1 accuracy of VGG-11 in Table 3 is 90.56%, indicating that there is still room for improvement in capturing inter-class differences.

In contrast, the confusion matrices for MobileNetV2 and DSCNet show higher classification accuracy. These two lightweight models fully leverage the efficient characteristics of depthwise separable convolutions, achieving strong classification performance in the majority of categories. However, for some complex scenarios or highly similar categories, these models still exhibit slight misclassification. The data in Table 3 shows that MobileNetV2 and DSCNet have Top-1 accuracies of 96.21% and 95.07%, respectively, demonstrating overall excellent classification performance.

Finally, the DSICBAM model performs the best in the confusion matrix, with almost no noticeable inter-class confusion. By incorporating the improved CBAM mechanism, Dropout regularization, and optimized weight distribution strategy, DSICBAM exhibits exceptional feature capture and classification capabilities, especially in complex scenarios. Its Top-1 accuracy (97.23%) and Top-5 accuracy (98.20%) are the highest, further proving the leading advantage of the DSICBAM model in various vehicle classification tasks.

In summary, the confusion matrix analysis confirms the superior performance of DSICBAM. It demonstrates strong robustness and generalization ability in classifying different vehicle categories, significantly outperforming the other comparison models.

**Fig 13:**
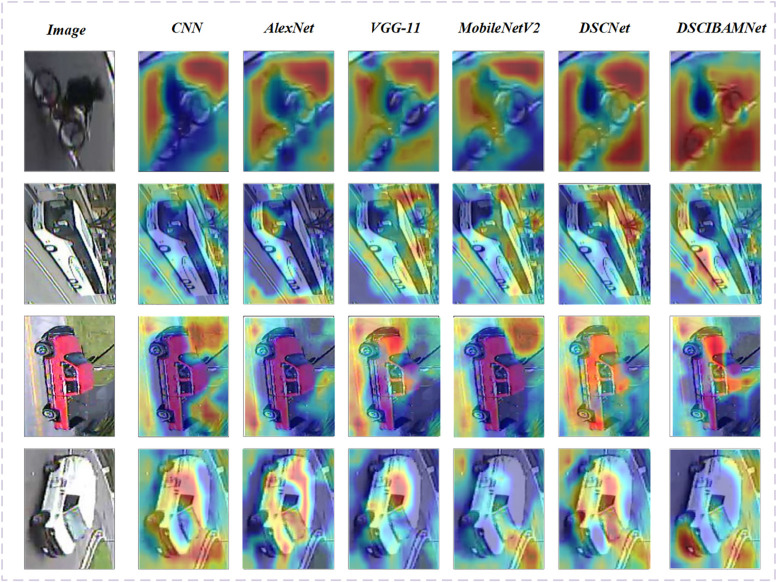
Gradcam visualization of different models.

To further analyze the models, Fig 13 presents the Grad-CAM [38] visualization results of different models, which are used to assess the models’ attention to key feature regions of the input images and their feature representation performance. From CNN to DSICBAM, the ability of the models to capture critical regions of vehicles gradually improves, with the distribution of prominent areas shifting from being scattered to more concentrated, showcasing a significant enhancement in feature learning capabilities.

The Grad-CAM visualization for the CNN model shows that its attention is mostly focused on the edges of the image and some irrelevant areas, with limited recognition of key vehicle parts such as logos and front ends. This indicates that shallow network structures struggle to effectively extract deep semantic features, leading to relatively poor model performance. AlexNet shows some improvement over CNN, with more target features covered by the prominent regions, but still includes some background interference, suggesting that its feature extraction capabilities contain some redundancy and have not fully focused on the critical areas.

The VGG-11 model’s deep structure significantly enhances feature extraction, with the Grad-CAM visualization showing the prominent regions concentrated around key vehicle parts, such as logos and front-end contours. However, due to its large number of parameters, its ability to fully utilize features in some scenarios is still insufficient. In contrast, MobileNetV2, with its lightweight design, is able to accurately capture the key features of vehicles while maintaining low computational complexity. Its Grad-CAM reveals that the prominent regions are mostly concentrated on important parts like logos and the front end, demonstrating a balance between performance and efficiency.

The DSCNet model, in the Grad-CAM visualization, shows a high focus on key vehicle features, with significantly reduced background interference. This is attributed to the combination of depthwise separable convolutions and attention mechanisms, which allow for efficient feature extraction with low computational cost. However, compared to DSICBAM, DSCNet still has room for improvement in capturing detailed features in complex scenarios.

Finally, the DSICBAM model performs the best in the Grad-CAM visualization. The prominent regions fully cover the critical vehicle parts, and the response to the background regions is minimal. This is due to the introduction of the improved CBAM module and dynamic weight allocation strategy, enabling the model to more effectively focus on important features, while the Dropout regularization enhances its adaptability to complex scenes.

In summary, the Grad-CAM visualizations reflect the gradual improvement in the models’ ability to focus on important features from shallow networks to DSICBAM. DSICBAM, with its enhanced attention mechanism and efficient feature learning, achieves the best vehicle classification performance, demonstrating significant advantages in both accuracy and generalization capabilities.

### Feature learning and results analysis of the model in this paper

**Fig 14:**
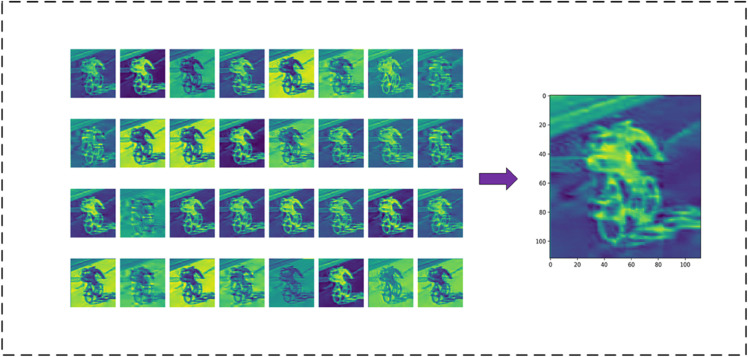
Network layer feature learning situation-Conv1 output features.

**Fig 15:**
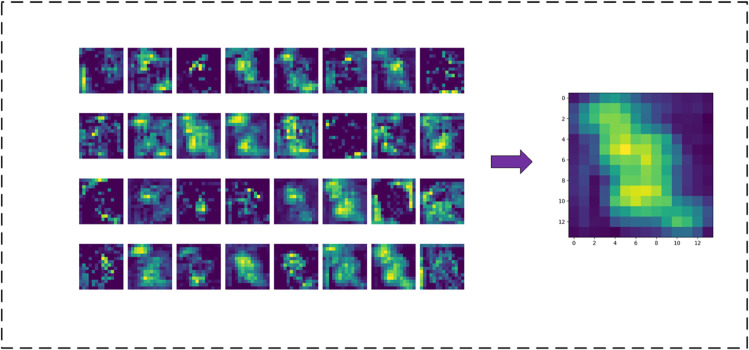
Network layer feature learning situation-DSICBAMBlock 4 output features.

To further analyze the feature learning ability of the model, we conducted a visualization analysis of the feature representations at different stages of the model. Specifically, we stacked the results of the 32 convolutional kernels at the Conv1 layer to obtain Fig 14, and similarly stacked the results of the 512 convolutional kernels in DSICBAMBlock 4 to obtain Fig 15. By comparing these two images, we can visually understand the model’s feature extraction and learning capabilities at different layers.

The Conv1 layer is the first convolution operation in the model, and its main task is to extract low-level features from the raw image. Fig 14 shows the feature map formed by stacking the effects of the 32 convolutional kernels in the Conv1 layer. From this, we can observe that the layer primarily captures low-level features such as edges, textures, and basic shapes in the image. These features serve as the foundation for extracting higher-level features in subsequent layers. Since the Conv1 layer has a relatively small number of convolutional kernels, the extracted features have a larger local scope, and the spatial resolution is still relatively high (112×112), allowing for the preservation of fine details in the input image.

DSICBAMBlock 4 is the fourth attention module in the model, and this module is responsible for further filtering and enhancing the extracted high-level features. In Fig 15, we can see that the feature map of this layer has a lower spatial resolution (14×14) but contains richer semantic information. The stacking of the 512 convolutional kernels indicates that DSICBAMBlock 4 can effectively capture the differences between categories, focusing on the most significant feature areas in the image, such as key vehicle parts (e.g., the front of the car, car logo, wheels). Furthermore, due to the introduction of the improved CBAM mechanism, the overall response of the feature map is more concentrated in meaningful areas, and background noise is significantly suppressed. The Dropout regularization further enhances the robustness of the module, enabling the model to more accurately extract multi-class features in complex scenarios.

By comparing Fig 14 and Fig 15, it is evident that as the depth of the model increases, the resolution of feature representations gradually decreases, while the richness of semantic information significantly increases. This hierarchical feature learning process fully reflects the inherent nature of deep learning models in extracting features from low-level to high-level. The features extracted by Conv1 focus more on global contours and edge information, while DSICBAMBlock 4 is better at focusing on key regions in the image, forming higher-level semantic features.

**Fig 16:**
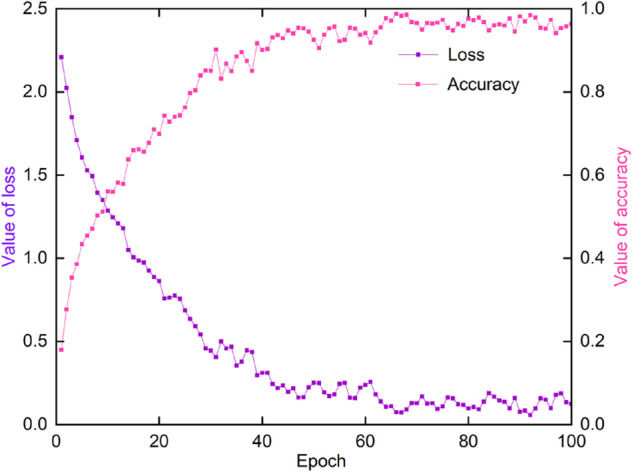
Accuracy and loss values during training process.

As shown in Fig 16, the accuracy and loss curves of the model during the training process illustrate its performance. From the initial stage to the 10th iteration, the loss quickly dropped from 2.21 to 1.28, demonstrating the model’s efficient feature learning ability in the early stages. As training progressed, the loss continued to decrease and eventually converged to a low range of 0.05-0.1 after 50 iterations, indicating the model’s stability and good convergence, successfully avoiding issues such as gradient explosion or divergence.

Meanwhile, the training accuracy quickly increased from the initial 18%, reaching about 85% after 50 iterations, and further climbed to a high level of 97%-98% in the later stages. This matches closely with the 97.36% average accuracy achieved in the testing phase. This result shows that the model demonstrates strong adaptability and generalization ability in learning the data distribution and features, mastering a large number of key features in a short period and continuously optimizing. The training process fully demonstrates the synergy of the improved CBAM and depthwise separable convolutions, enabling the model to quickly capture core features of vehicle images in the early stages while steadily improving accuracy and reducing the loss value in later stages through regularization and attention mechanism optimization.

Overall, the model demonstrates high efficiency in feature learning and continuous optimization potential during training. The final training accuracy stabilized at 97%-98%, indicating that its adaptability to complex traffic scenes and multi-class vehicle features is well-developed. This foundation played a key role in achieving high accuracy in the testing phase and further validates the model’s feasibility and reliability in real-world applications.

**Fig 17:**
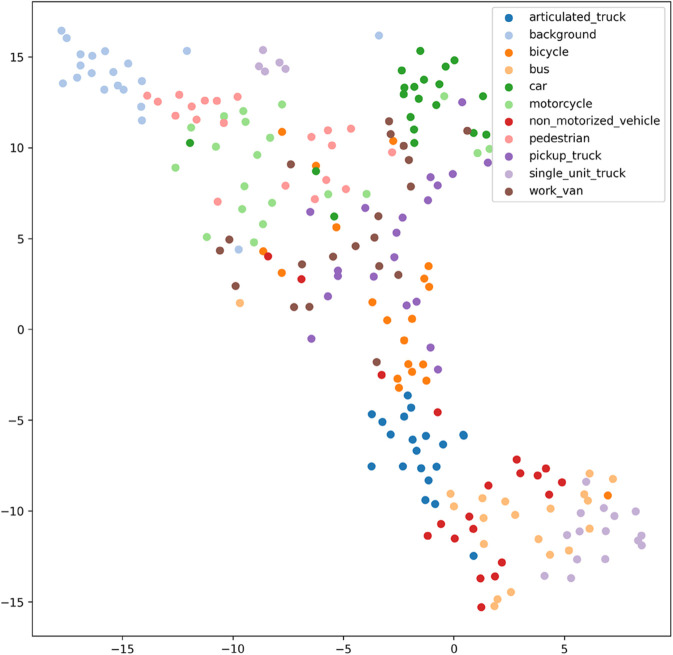
Clustering of the model in different network layers-CNN.

**Fig 18:**
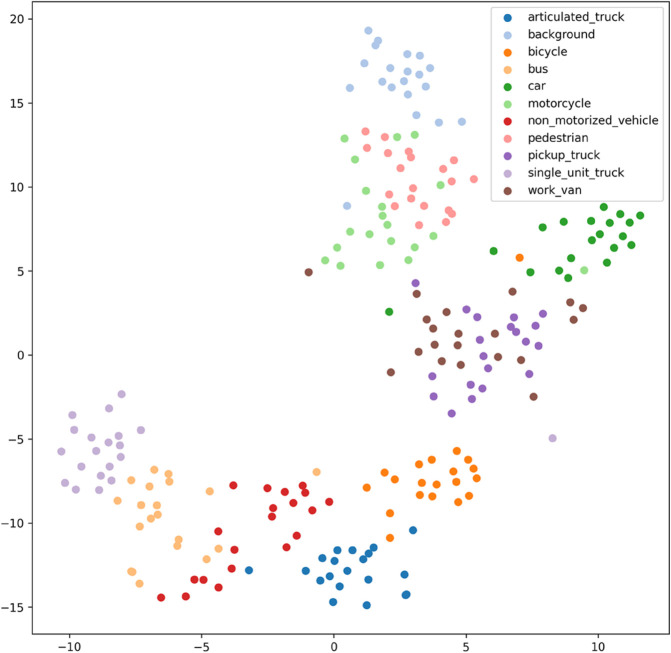
Clustering of the model in different network layers-DSICBAMBlock 1.

**Fig 19:**
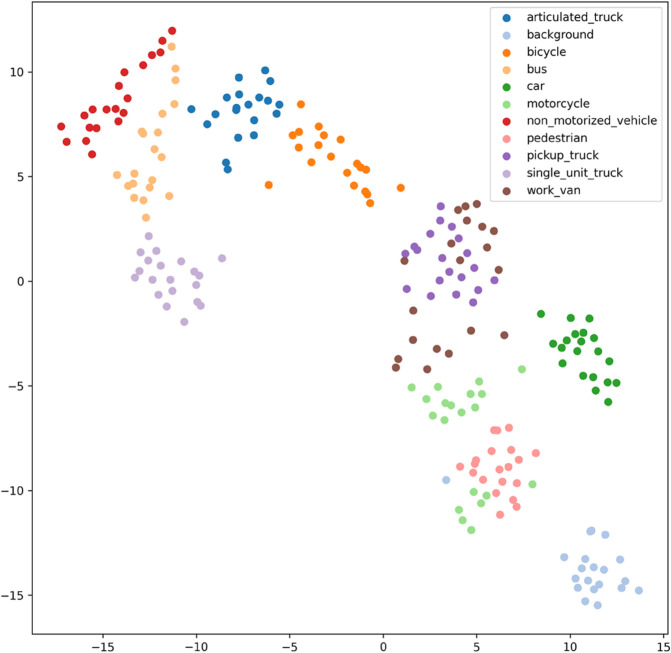
Clustering of the model in different network layers-DSICBAMBlock 2.

**Fig 20:**
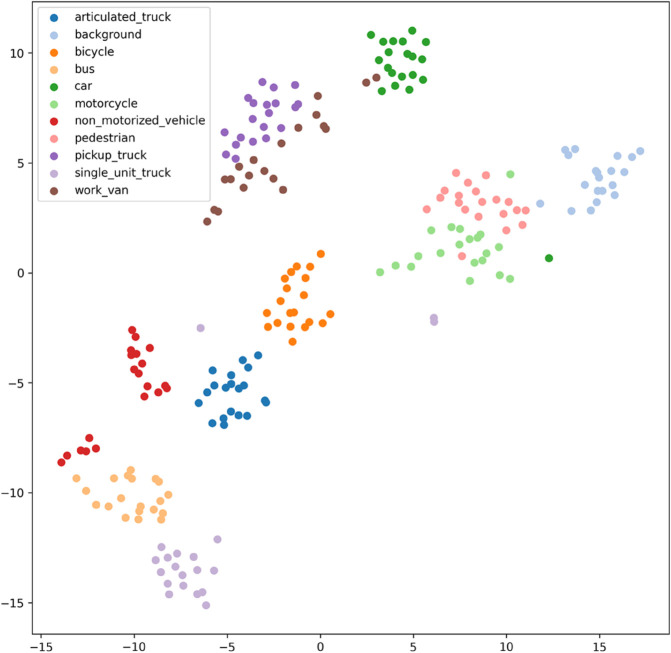
Clustering of the model in different network layers-DSICBAMBlock 3.

**Fig 21:**
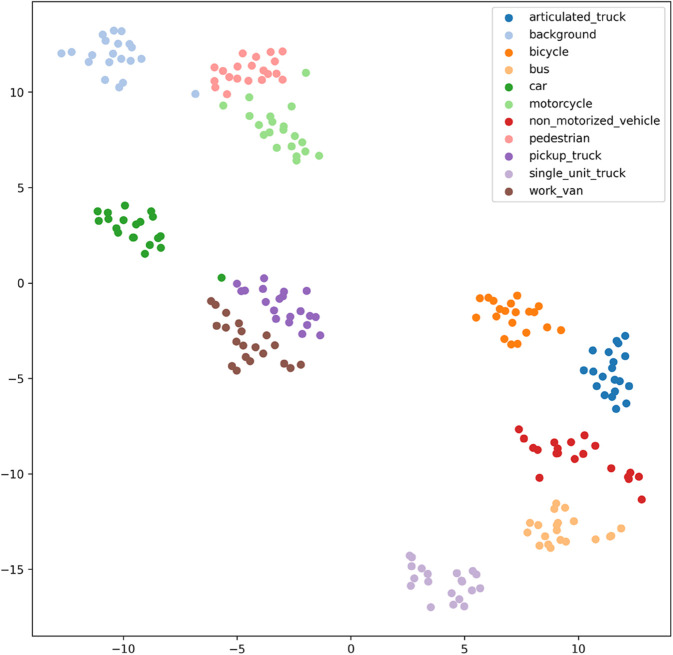
Clustering of the model in different network layers-DSICBAMBlock 4.

**Fig 22:**
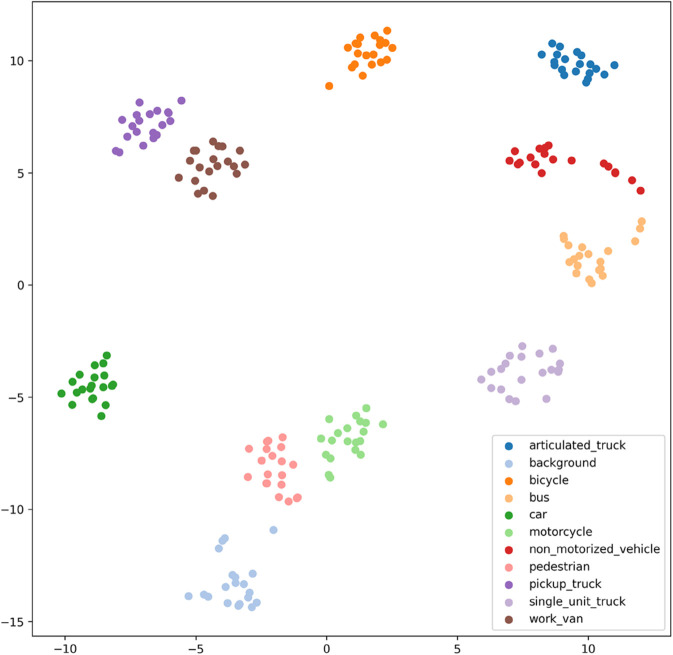
Clustering of the model in different network layers-FC.

Fig 17, Fig 18, Fig 19, Fig 20, Fig 21, and Fig 22 display the t-SNE clustering results of the model at different network layers, including the initial convolutional layer (Conv1), the four DSICBAMBlocks, and the final fully connected layer (FC). Overall, as the network depth increases, the model’s ability to recognize and distinguish vehicle features significantly improves. The Conv1 layer can only extract low-level edge and texture information, with category clusters showing significant overlap. However, after the introduction of the improved CBAM mechanism in the DSICBAMBlocks, the point clouds of different categories gradually separate in the high-dimensional space, and the class boundaries become clearer. By the fourth DSICBAMBlock, the model has acquired a high level of semantic representation ability, with nearly no overlap between categories, indicating that the depthwise separable convolution and improved attention mechanism work synergistically in multi-scale feature extraction and focusing on key regions, significantly enhancing the discriminative power of feature representation. Finally, the FC layer integrates the high-dimensional features and maps them to the category space, showing highly compact category clusters and distinct inter-class boundaries, suggesting that the model has effectively utilized high-level semantic features, greatly improving classification accuracy and stability.

The multi-level feature clustering analysis provides a clear visual representation of the model’s efficiency and accuracy in the vehicle classification task. From initial shallow edge detection to precise separation of deep semantic features, the model gradually refines its understanding and representation of vehicle categories at each layer. It is worth emphasizing that the model achieved an average accuracy of 97.36% on the test set, further confirming the effectiveness of the improved CBAM module within the depthwise separable convolution framework. This attention mechanism, with its parallel fusion in both the channel and spatial dimensions and the introduction of Dropout regularization, provides strong support for feature extraction and redundant information filtering in complex scenarios. Future research could focus on dynamic weight distribution in the attention mechanism, multi-scale feature capture, and adaptability to cross-domain data, offering more robust and applicable solutions for vehicle classification and broader traffic vision tasks.

### Validation on different datasets

To further evaluate the model’s generalization capability and practical application potential, we conducted comparative validation experiments across different datasets. While the initial experiments demonstrated strong classification performance on a relatively small dataset, the limited dataset scale may not fully reflect the model’s adaptability and stability in more complex scenarios.

#### Stanford cars datasets.

To further validate the model’s generalization capability and practical applicability, we introduced the larger and more diverse Stanford Cars dataset. This dataset includes 16,185 high-resolution images across 196 vehicle categories, characterized by real-world scenarios, diverse lighting conditions, complex backgrounds, and fine-grained classification challenges. These features make it highly valuable for testing and validation.

For this experiment, we selected five representative and challenging categories from the Stanford Cars dataset: SUV, Sedan, Sports Car, Coupe, and Convertible, comprising a total of 5,300 images. These categories not only cover common vehicle types such as SUV and Sedan but also include high-similarity categories like Sports Car and Coupe, as well as relatively unique types like Convertible. This selection balances inter-category diversity and experimental complexity, laying a solid foundation for analyzing the model’s feature extraction capability and generalization performance. The specific results can be seen in Table 4.

**Table 4:** Selected Stanford cars dataset.

Category	Description	Total Samples	Training Samples	Testing Samples
SUV	Sport Utility Vehicle	1060	848	212
Sedan	Sedan	1040	832	208
Sports Car	Sports Car	1040	832	208
Coupe	Two-door Car	1080	864	216
Convertible	Convertible Car	1080	864	216
**Total**	—	**5300**	**4240**	**1060**

*Table notes:* This table presents the category-wise distribution of the selected Stanford Cars dataset, detailing the total, training, and testing samples.

The dataset was divided into a training set and a testing set, with 4,240 images in the training set and 1,060 images in the testing set. During data preprocessing, all images were resized to a resolution of 224×224. Data augmentation techniques such as random cropping, horizontal flipping, and brightness adjustment were applied to enhance the model’s generalization ability. The experiments were conducted under the same hardware and software environment to ensure the reproducibility and reliability of the results.

Through this comparative validation, we aim to comprehensively evaluate the model’s classification performance in diverse scenarios, particularly its ability to distinguish between similar categories in fine-grained classification tasks. Additionally, the experiment provides insights into the model’s adaptability to complex backgrounds and varying lighting conditions. The final results are expected to enhance the credibility of the study and provide critical directions for future improvements and theoretical advancements.

#### Test results from different methods.

Table 5 summarizes the performance of the DSICBAM model compared to CNN, AlexNet, VGG-11, MobileNetV2, and DSCNet on the Stanford Cars test set. The results demonstrate that DSICBAM significantly outperforms the other models across multiple key metrics. Specifically, DSICBAM achieved a mean accuracy of 96.51%, precision of 96.14%, recall of 96.35%, F1-score of 96.24%, Top-1 accuracy of 96.53%, and Top-5 accuracy of 97.23%. Notably, its mean accuracy and Top-1 accuracy exceeded the second-best-performing MobileNetV2 by 2.39 and 2.62 percentage points, respectively, highlighting its superior feature extraction capabilities and strong generalization performance.

**Table 5:** Test results of different methods.

Model	Avg Accuracy (%)	Precision (%)	Recall (%)	F1-score (%)	Top-1 Accuracy (%)	Top-5 Accuracy (%)
CNN	85.14	84.67	84.78	84.72	84.81	90.23
AlexNet	90.23	89.85	90.14	89.99	90.12	94.02
VGG-11	91.56	91.14	91.38	91.26	91.42	95.21
MobileNetV2	94.12	93.68	93.82	93.75	93.91	96.43
DSCNet	93.78	93.12	93.45	93.28	93.35	95.67
DSICBAM (ours)	96.51	96.14	96.35	96.24	96.53	97.23

*Table notes:* This table shows the test results of different methods across various metrics.

Compared to the initial test results on the original MIO-TCD dataset, the larger scale and finer granularity of the Stanford Cars dataset further validate the suitability and robustness of DSICBAM in more complex task scenarios. Despite the increased data volume and category complexity, DSICBAM maintained a high level of performance, whereas other models showed varying degrees of fluctuation. For example, CNN and AlexNet only achieved Top-1 accuracies of 84.81% and 90.12%, respectively, revealing limitations in their feature extraction capabilities. In contrast, DSICBAM leveraged its enhanced CBAM module and depthwise separable convolutions to effectively capture spatial and channel features while reducing computational costs, ensuring consistent performance superiority.

Moreover, while lightweight models like MobileNetV2 and DSCNet exhibited relatively close performance on the Stanford Cars dataset, DSICBAM retained a leading edge through more efficient feature extraction and a finely tuned module design. Overall, DSICBAM demonstrated exceptional performance not only on smaller datasets but also on larger, more challenging datasets, showcasing its broad applicability and reliability, thereby providing strong support for its adoption and optimization in practical applications.

A comparison of the test results on the MIO-TCD and Stanford Cars datasets reveals the performance differences of DSICBAM under varying dataset scales. On the smaller MIO-TCD dataset, DSICBAM achieved a Top-1 accuracy of 97.36%, significantly outperforming other models, reflecting its efficient feature extraction and classification capabilities in data-scarce environments. On the Stanford Cars dataset, despite the significant increase in sample size and task complexity, DSICBAM’s Top-1 accuracy decreased by only about 0.85 percentage points, demonstrating excellent generalization and robustness. In contrast, other models, such as MobileNetV2, showed more noticeable performance fluctuations, with its Top-1 accuracy dropping from 96.85% to 94.12%.

In summary, DSICBAM’s consistently superior performance on both small and large datasets underscores its compatibility and adaptability. It effectively and accurately classifies data across various scales and complexities, making it particularly suitable for scenarios requiring a balance of computational efficiency and classification precision.

#### T-SNE clustering analysis.

**Fig 23:**
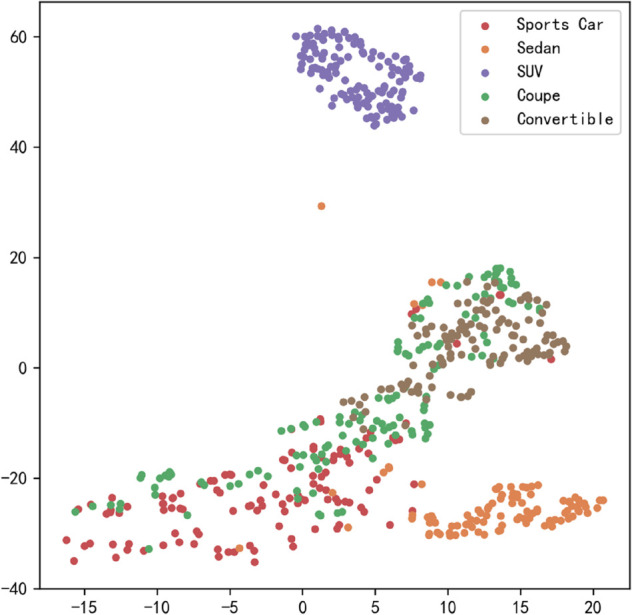
Clustering of different comparative models on the Stanford Cars dataset-CNN.

**Fig 24:**
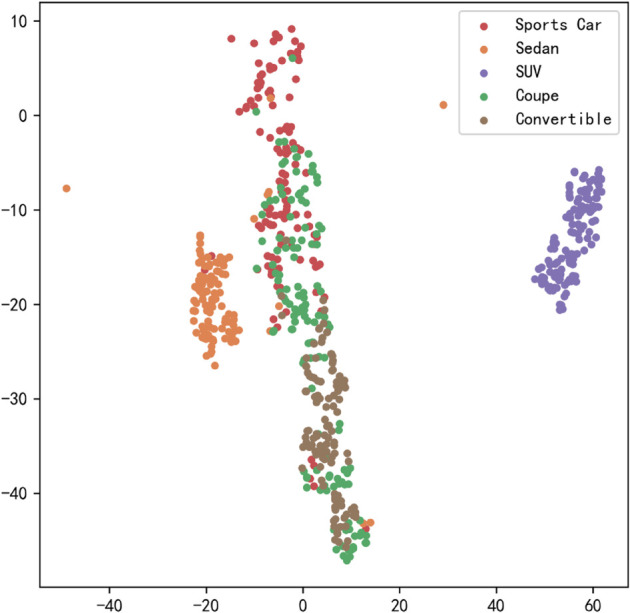
Clustering of different comparative models on the Stanford Cars dataset-AlexNet.

**Fig 25:**
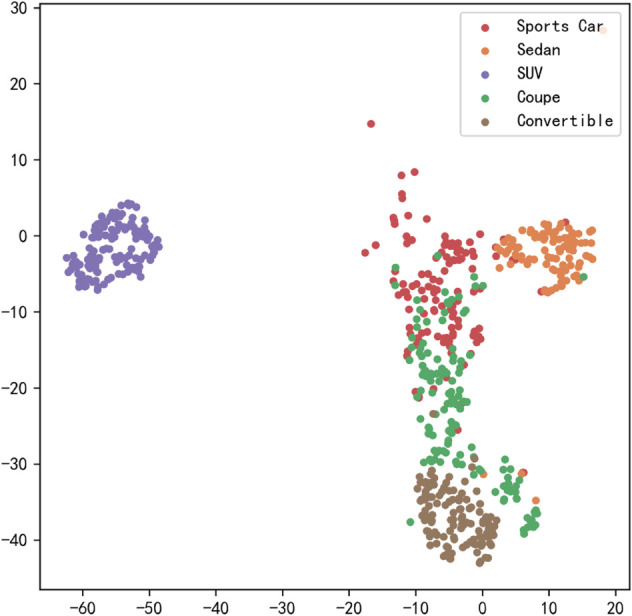
Clustering of different comparative models on the Stanford Cars dataset-VGG-11.

**Fig 26:**
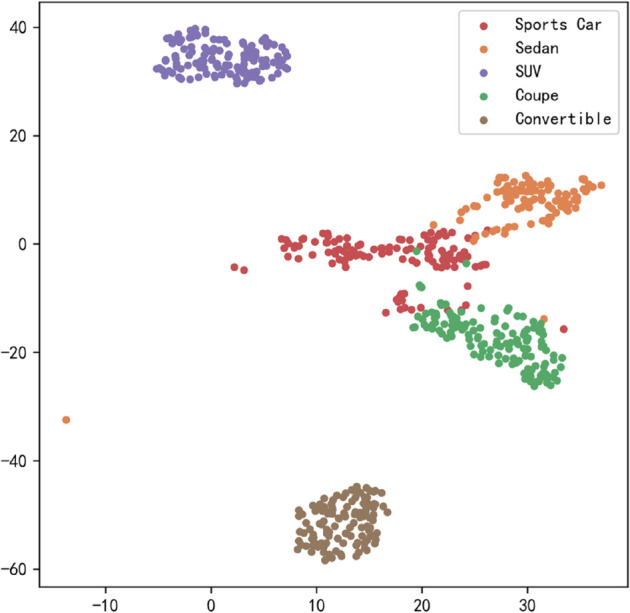
Clustering of different comparative models on the Stanford Cars dataset-MobileNetV2.

**Fig 27:**
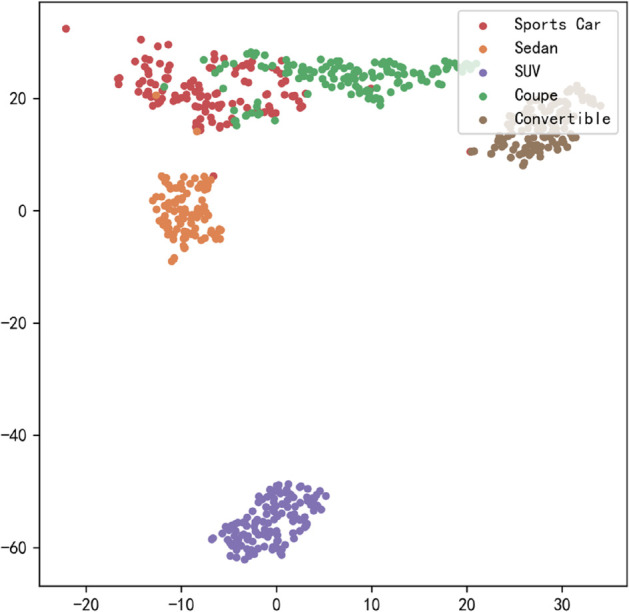
Clustering of different comparative models on the Stanford Cars dataset-DSCNet.

**Fig 28:**
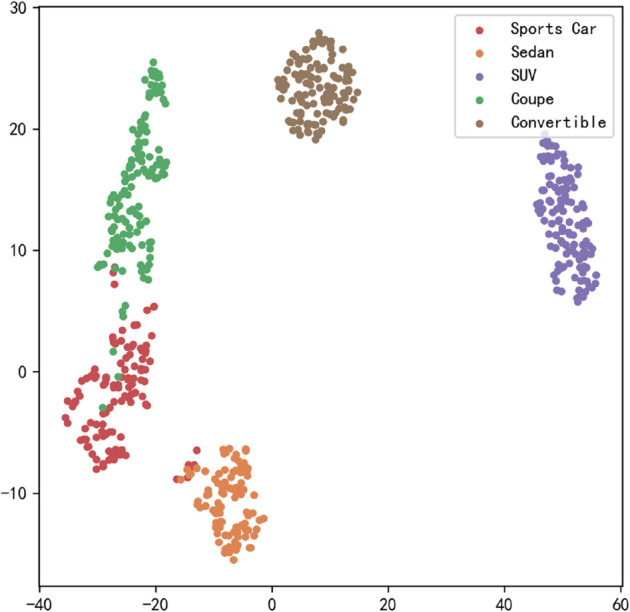
Clustering of different comparative models on the Stanford Cars dataset-DSICBAM.

Fig 23, Fig 24, Fig 25, Fig 26, Fig 27, and Fig 28 present the T-SNE clustering analysis results of different comparative models on the Stanford Cars dataset. By visualizing the spatial distribution of high-dimensional features, it intuitively reflects the differences in each model’s ability to extract features and distinguish categories.

From the clustering results, the CNN model shows a relatively chaotic distribution of category features, with blurred boundaries between categories and scattered intra-class features, indicating a significant lack of feature extraction capability, which corresponds to its Top-1 accuracy of only 84.81%. Compared to CNN, AlexNet shows some improvement in clustering, with partial category boundaries beginning to emerge, but there is still considerable overlap between classes, suggesting limited ability to capture fine-grained features.

VGG-11 further optimizes the feature distribution, with clearer boundaries between most categories and more compact intra-class features. However, a few instances of overlap among neighboring categories remain, indicating room for improvement in fine-grained classification tasks. As a lightweight model, MobileNetV2 demonstrates good category separation ability in the T-SNE plot, with clear category boundaries and tight feature distributions. This aligns with its Top-1 accuracy of 94.12%, reflecting strong generalization performance.

DSCNet’s feature distribution is close to that of MobileNetV2, but some categories still exhibit overlapping high-dimensional features, revealing limitations in its feature extraction module design. In contrast, the DSICBAM model’s T-SNE clustering results show a marked advantage in category separation, with clear boundaries and compact feature distributions, and virtually no overlap between categories. This fully demonstrates its outstanding performance in extracting fine-grained features and distinguishing categories, which is highly consistent with its mean accuracy of 96.51% and Top-1 accuracy of 96.53%.

Overall, the T-SNE clustering analysis in Fig 17, Fig 18, Fig 19, Fig 20, Fig 21, and Fig 22 further validates the feature extraction capabilities of each model on a complex dataset. In particular, the DSICBAM model exhibits excellent applicability with its more efficient feature extraction and classification abilities, providing strong support for its promotion in practical applications.

### Ablation experiment

Table 6 presents the average accuracy and testing time of the DSICBAM model and its various ablation configurations on the MIO-TCD and Stanford Cars datasets. The results show that the complete DSICBAM model demonstrates outstanding performance on both datasets, achieving an average accuracy of 97.36% on the MIO-TCD dataset and 96.51% on the Stanford Cars dataset, while maintaining relatively low testing times of 12.42 seconds and 15.82 seconds, respectively.

**Table 6:** Results of ablation experiments on different datasets.

Data Set	MIO-TCD		Stanford Cars	
Evaluating Indicator	Avg Accuracy (%)	Test Time (s)	Avg Accuracy (%)	Test Time (s)
Dsicbam	97.36	12.42	96.51	15.82
Remove CBAM Module	96.21	11.71	94.85	14.94
Remove Depthwise Separable Convolution	95.48	18.65	93.78	20.40
Remove All Enhancement Modules	92.15	19.33	85.14	21.73

*Table notes:* The table summarizes ablation results, comparing models with and without enhancement modules on MIO-TCD and Stanford Cars datasets.

The ablation experiments indicate that removing the CBAM module causes the average accuracy to drop to 96.21% and 94.85%, respectively, highlighting the critical role of the CBAM module in enhancing channel and spatial features. Removing the depthwise separable convolution further reduces the average accuracy to 95.48% and 93.78%, while significantly increasing testing time, demonstrating the importance of depthwise separable convolution in improving model efficiency and maintaining high accuracy.

When all enhancement modules are removed, the model’s performance declines sharply, with average accuracies of 92.15% and 85.14% on the MIO-TCD and Stanford Cars datasets, respectively, and testing times rising to 19.33 seconds and 21.73 seconds. This further verifies the contribution of the enhancement modules to the overall model performance.

It is noteworthy that the Stanford Cars dataset, due to its higher complexity and fine-grained classification tasks, results in lower average accuracies across all model configurations compared to the MIO-TCD dataset, especially for models with weaker configurations, which show more significant declines. This indicates that the DSICBAM model, through the collaborative optimization of the CBAM module and depthwise separable convolution, possesses stronger feature extraction capabilities and generalization performance, enabling it to maintain excellent classification performance in more complex task scenarios while achieving both lightweight and efficiency. These results further confirm the rationality and applicability of the DSICBAM design.

### Comprehensive analysis

A deeper evaluation of the overfitting risk potentially caused by small datasets can reveal the applicability and potential limitations of the DSICBAM model in specific tasks. In the small dataset MIO-TCD experiments, DSICBAM achieved an average accuracy of 97.36%, demonstrating excellent classification ability. However, the characteristics of small datasets may lead the model to memorize specific data patterns during training rather than generalize to a broader data distribution, which is the overfitting risk. Specifically, the limited diversity of samples in small datasets may not fully cover the complex scenarios in the target task, causing the high accuracy on the test set to reflect more the memorization of the training set rather than the actual generalization performance.

On the larger and more complex Stanford Cars dataset, DSICBAM’s average accuracy slightly decreased to 96.51%, indicating the model still maintains strong robustness in more challenging tasks. However, the removal of certain modules in ablation experiments led to significant performance declines, further indicating that some enhancement modules may help alleviate the overfitting issue to some extent. Future research can explore the following directions: 1) Enhancing data diversity: improving the model’s generalization by applying data augmentation techniques or generating more diverse samples using generative adversarial networks. 2) Optimizing regularization strategies: introducing stronger regularization methods (such as Dropout, weight decay) or adjusting training sample weight distribution to reduce overfitting risk. 3) Lighter network structures: further simplifying the network architecture while maintaining high performance to reduce reliance on specific sample features.

Moreover, future studies should pay more attention to the performance of DSICBAM in practical application scenarios, especially in tasks with limited data but complex scenes. By continuously optimizing model architecture, designing more efficient feature extraction modules, and developing new training strategies, DSICBAM can further reduce the overfitting risk caused by small datasets and expand its applicability in diverse tasks.

## Conclusion

This paper proposes a lightweight model called DSICBAM, which combines depthwise separable convolution with an improved attention mechanism, successfully balancing classification accuracy and computational efficiency. Experimental results demonstrate that DSICBAM outperforms multiple comparative models, achieving an average accuracy of 97.36% in the MIO-TCD experiments, while maintaining strong generalization ability and stability on the larger-scale Stanford Cars validation dataset. Grad-CAM-based visualization analysis further validates its ability to focus on critical regions, providing strong support for classification tasks in complex scenarios.

(1) DSICBAM innovatively integrates depthwise separable convolution with an improved CBAM module, significantly reducing computational complexity while substantially improving classification accuracy. It comprehensively surpasses traditional models such as CNN, AlexNet, and VGG-11 in key metrics including average accuracy, Top-1, and Top-5 accuracy, achieving an excellent balance between efficiency and precision.

(2) The model performs especially well in resource-constrained scenarios, demonstrating strong practicality with efficient feature extraction capabilities and low computational cost. Although there remains room for improvement under extreme lighting conditions or highly similar categories, DSICBAM’s performance on validation datasets proves its good generalization ability and robustness.

(3) Future research will focus on combining model compression techniques such as quantization and pruning, and formulating specific deployment strategies for embedded devices and mobile terminals, further enhancing the model’s operational efficiency and adaptability. Meanwhile, exploring multi-task learning frameworks to strengthen the model’s generalization ability and capacity to handle larger, more diverse datasets will also be pursued.
